# Late Onset Ornithine Transcarbamylase Deficiency Triggered by an Acute Increase in Protein Intake: A Review of 10 Cases Reported in the Literature

**DOI:** 10.1155/2020/7024735

**Published:** 2020-04-25

**Authors:** E. Barkovich, A. L. Gropman

**Affiliations:** ^1^Department of Radiology, George Washington University School of Medicine and Health Sciences, Washington, DC, USA; ^2^Department of Neurology, George Washington University School of Medicine and Health Sciences, Children's National Medical Center, Washington, DC, USA

## Abstract

While the urea cycle disorders (UCDs) classically present in the neonatal stage, they have become increasingly recognized as a rare cause of unexplained hyperammonemic encephalopathy in adults. Many metabolic triggers for late-onset UCDs have been described in the literature including excessive protein intake. In this case series, ten such documented cases are reviewed with analysis of patient demographic, protein load, treatment course, and patient outcome. Common delays in treatment include recognition of hyperammonemia as the cause of encephalopathy and initiation of hemodialysis. In only one case was a diet history used to raise suspicion for a metabolic derangement. Metabolic disorders remain an important consideration in adults presenting with encephalopathy not explained by more common etiologies, and recent and remote dietary history may provide valuable information.

## 1. Background

The urea cycle consists of six enzymes and two transporters which produce urea from ammonia within the mitochondria and cytosol of hepatocytes. A mutation to any of these proteins may cause a urea cycle disorder (UCD) in which the body's ability to process and excrete nitrogen-containing molecules is impaired and toxic ammonia builds up. The incidence of the UCDs is estimated to be 1 : 35,000 births; partial defects which remain undiagnosed due to mild symptoms or incomplete investigation likely mean the true figure is substantially higher [1]. Ornithine transcarbamylase (OTC) is the enzyme most commonly involved due to its X-linked pattern of inheritance, affecting 1 : 56,000 births.

The UCDs classically present in the neonatal stage with signs of hyperammonemia (HA) such as lethargy and vomiting, progressing to seizures, coma, and death. The UCDs have also been identified as a cause of encephalopathy in young and middle-aged adults with partial enzymatic deficiency. These episodes present sporadically when normal nitrogen metabolism is disturbed by physiologic events such as acute illness, surgery, the postpartum period, or menstruation. Patients are often entirely asymptomatic prior to presentation but may have a history of learning disabilities and behavioral difficulties [2]. Other documented triggers of HA include valproic acid [3], total parenteral nutrition (TPN) [4], corticosteroids [5], gastric bypass surgery [6], GI bleeding [7], malnutrition [8], and protein loading. We present a review of ten documented cases in which increased protein intake likely triggered HA in previously undiagnosed individuals with partial ornithine transcarbamylase deficiency (OTCD). The importance of regulating caloric and protein intake has long been a cardinal component of UCD management, and the proclivity of both undiagnosed and heterozygote females for low-protein diets has been documented [6]. This is the first case series that explicitly studies nutritional triggers in the late-onset population. We again emphasize the relevance of an appropriate diet history in patients with possible metabolic disorders as well as the importance of considering an inborn error of metabolism in any patient presenting with altered mental status following a major dietary change.

## 2. Materials and Methods

Thorough literature searches of PubMed and Google Scholar were conducted using search terms including “late-onset” “urea cycle disorder,” “ornithine transcarbamylase,” and “high protein.” Similar keywords were used in a Google search in order to find nonpublished reports such as poster presentations and nonmedical accounts published in newspapers. Supplemental details of the cases are provided in Supplementary Materials.

## 3. Results

Ten cases of late-onset HA likely due to a recent increase in protein were documented between January 2003 and August 2017. Of these patients, eight were male and two were female with an average age of 34 (range of 17–59) at presentation. Eight were diagnosed with OTCD, one patient was presumed to have OTCD based on history, and the final patient had an unspecified UCD (of note the patient has a healthy 5-year-old son). Of the eight OTCD diagnoses, 2 were presumed based on characteristically abnormal amino acid levels, while 6 were confirmed with DNA sequencing. Four patients died, one survived with lasting neurocognitive deficits, and five survived without noted cognitive change. The cases are summarized below, and a synopsis is available in Supplementary Materials.

 
*Case 1.* Gaspari et al. [9]: 32-year-old Italian female presented with a generalized tonic-clonic seizure. She had had multiple psychiatric evaluations and had been previously diagnosed with epilepsy, and had recurrent episodes of vomiting and lethargy over the past 10 years. Although she had a history of meat refusal, her parents reported that she had eaten a “high quantity of meat on two consecutive occasions” prior to presentation.
 
*Case 2.* Panlaqui et al. [10]: 48-year-old Australian male body builder presented following three days of flu-like symptoms with increasing lethargy. He had no medical conditions but was described as having a six month decline in memory. In the emergency room, he was found to have asterixis. His somnolence soon progressed to loss of consciousness requiring intubation.
 
*Case 3.* Ben-Ari et al. [11]: 47-year-old Israeli male presented with vomiting, intermittent lethargy, and increasing somnolence over three days. In the hospital, his condition worsened to coma requiring mechanical ventilation. He had no notable medical history. On hospital day 4, the care team learned that he had started on a low-carbohydrate (<20  g/day), unlimited high-protein, and high-fat Atkins diet 3 days prior to presentation.
 
*Case 4.* Thurlow et al. [12]: 24-year-old British male presented with nausea and vomiting for 1 week. He was found to have prolonged clotting tests, otherwise labs were normal. After IV hydration, he felt improved enough to be discharged the next day, but returned 3 days later with vomiting and disorientation. His past history was significant for ADHD unresponsive to methylphenidate and severe headaches with facial twitching at age 11. Recently, he had become concerned about his body image and had increased his meat intake, started taking food supplements, and started using methandrostenolone and creatine.
 
*Case 5*. ABC [13]: 20-year-old Canadian male passed away following brief illness after feeling progressively ill for several weeks. He was a junior hockey player who had been on a high-protein eating plan in an attempt to boost muscle mass.
 
*Case 6*. Telegraph [14]: 50-year-old British male presented with vomiting and lethargy progressing to coma requiring mechanical ventilation. He had no past medical history but had started the high-protein Dukan diet 2.5 days earlier.
 
*Case 7*. Choi et al. [15]: 59-year-old Korean male presented with progressive lethargy and confusion since that morning. During childhood, he had recurrent episodes of abdominal pain and convulsions but none recently. Family reported episodes of nausea and vomiting 3 days prior after eating a large amount of dog meat and 2 days prior after eating chicken and freshwater snails.
 
*Case 8*. Rush et al. [16]: 20-year-old American male found unresponsive following 2 months of weight loss, nausea, and vomiting. He had been taking a protein supplement, creatine, arginine supplement, and stanozolol for several months as part of a strength-training program.
 
*Case 9*. Alameri et al. [17]: 17-year-old white (Emirati) male presented with 6 minute generalized tonic-clonic seizure following a week of generalized malaise with intermittent vomiting without fever, chills, diarrhea, or shortness of breath. He had started on a high-protein supplement 1 week prior to developing these symptoms.
 
*Case 10*. Perth Now [18]: 25-year-old Australian female found unconscious in her apartment. She was taking protein shakes and eating protein-rich foods to get in shape for a body-building competition. She had complained of lethargy and feeling “weird” earlier that month.

See supplemental info for hospital course, lab values, and patient outcomes.

## 4. Discussion

Hyperammonemia is a medical emergency and requires aggressive therapy. This may include dialysis as well as implementation of ammonia scavengers and protein restriction in the diet. Giving adequate nonprotein calories is important to prevent catabolism. The medical management is complex, and early involvement of a metabolic expert is imperative. Hyperammonemic encephalopathy is an important cause to consider in any adult presenting with encephalopathy or unexplained vomiting and lethargy due to the life-threatening sequelae of cerebral edema, herniation, and death. A measurement of plasma ammonia is always indicated and turnaround time may be as short as 20 minutes [19]. Given normal liver synthetic function and no prior history of liver disease, an inborn error of metabolism is likely. While a family history may be suggestive and penetrance of the trait is complete, variability of environmental stressors allow the partial UCDs to present at any point in the lifespan (if at all) [20]. De novo mutations are far more common in neonatal onset disease, but may also occur in partial enzymatic deficiency. Furthermore, in female heterozygotes with partial OTCD, nonrandom *X* inactivation may complicate attempts to predict disease timing and severity.

A brief review of protein metabolism explains how protein loading, especially when coupled with carbohydrate restriction as seen with the Atkins and Dukan diets, can exacerbate an underlying UCD. While the body stores carbohydrates and lipids as glycogen and triglycerides, it does not have a storage form for amino acids. Amino acids surplus to current synthetic requirements must be deaminated to pyruvate, acetyl-CoA, or acetoacetyl-CoA for use in the citric acid cycle or anabolism. The amino groups liberated from these reactions are transported to the liver in the form of glutamate or glutamine for conversion into urea. In individuals with partial UCDs, a significant increase in protein/amino acid substrate can disturb the delicate kinetics of the urea cycle, leading to elevated levels of glutamine and ammonia. When dietary carbohydrate intake is reduced, protein catabolism and amino acid deamination is favored, further raising plasma ammonia levels.

Similar to the prevalence of the disease itself, the frequency of protein loading as a trigger for HA in partial UCDs is unclear. While these are the only ten published cases our search was able to find, there are almost certainly more cases that go unrecognized or unpublished. In fact, in the Telegraph article, metabolic specialist Dr. Yosof Rahman refers to two cases in which high-protein diets led to a coma; while one of these cases was previously reported by Thurlow and included in our study, the other involving a 24-year old was never published. Further, clouding the picture is the reality that the trigger of late-onset HA is often multifactorial. In Panlaqui et al, the 48-year-old man's flu-like symptoms may have been due to rising ammonia from protein supplements for body-building but also could be explained by an unrelated viral infection which may have contributed to his metabolic imbalance. Likewise in Thurlow et al. and Rush et al., the men had also recently began taking anabolic steroids and creatine in addition to protein supplementation.

### 4.1. Medical History

Previous symptoms suggestive of unidentified and untreated periods of HA (memory changes, attention deficits, seizures in childhood, periods of vomiting and lethargy, etc.) were present in six of the ten patients. These findings could indicate reduced enzymatic function relative to the others, increased environmental stressors, or be explained by an independent medical condition. Previous history was not found to be related to acute outcome as only two of the four fatalities had prior symptoms of HA. While the sample size of ten has insufficient statistical power, it seems feasible that the most significant predictors for acute patient outcome were the size and timing of the protein bolus relative to degree of enzymatic dysfunction and the promptness of diagnosis and initiation of appropriate treatment.

### 4.2. Diagnosis

In reviewing these cases, two common themes of uncertainty were identified: (1) the identification of HA as the cause of encephalopathy and (2) timing of hemodialysis (HD) for treatment of HA. Unsurprisingly, an appropriate history and physical exam remain an excellent starting point; Panlaqui et al. detected asterixis on exam and pursued a possible metabolic cause. While this finding has unknown specificity and sensitivity, it remains a useful tool for any resourceful clinician [21]. The history of an encephalopathic patient requires consultation and collateral information from family. Dietary habits may be especially difficult to glean in this context, and recent dietary history may be altogether impossible in cognitively altered patients who live alone. Only one of the patients in this study had their treatment direction influenced by dietary history, despite the fact that dietary changes were identified afterwards as the primary trigger in nearly all cases. Gaspari et al. were able to use collateral information from the parents and the patient's prior nonspecific symptoms to direct them in a metabolic direction. In two other cases, EEG findings suggested a metabolic cause, while in four others, ammonia tests were ordered empirically as part of the initial work-up for unspecified encephalopathy. Thurlow et al. did not identify HA as the cause of encephalopathy until amino acid results returned post-mortem.

### 4.3. Treatment

In hyperammonemic encephalopathy, dialysis should be first line treatment even without definitive diagnosis due to the rapidly toxic effects of ammonia [1]. Although reducing levels to <150  *µ*M is usually sufficient, NH_3_ should continue to be monitored due to the frequency of rebound [1]. Unlike with hepatic encephalopathy, a noninherited cause of HA, lactulose is not recommended as a first line treatment for UCDs as it must reach the colon to begin to take effect, and relies upon regular bowel movements for ammonia excretion. Despite this recommendation, four patients received lactulose and experienced clinical worsening with increases in plasma ammonia levels. Although HA had been identified, it was only after poor response that clinicians considered HD and ordered further amino acid analysis to investigate an inherited metabolic cause. These attempts to use lactulose and avoid the invasive nature of dialysis demonstrate both the rarity and general lack of familiarity with late-onset presentations of inborn errors of metabolism. Given the high mortality rate of late-onset presentations, further education is needed concerning appropriate escalation of treatment.

The inclusion of two patients with anabolic steroid use was the cause of significant debate among the writers of this paper. From a metabolic perspective, anabolic steroids promote protein synthesis and therefore should reduce amino acid deamination and discourage ammonia build-up. Anabolic steroids are known to be hepatotoxic and can cause liver injury in both the subacute and chronic setting [22]. Further study is necessary to explore their aggregate effects on the urea cycle; however, given the known adverse effects of anabolic steroids, this seems unlikely to occur.

### 4.4. Diagnosis

Genetic and enzyme analysis are the gold standard for diagnosis but are not currently feasible for the acute setting. Identification of a UCD is usually based on the metabolic footprint of the dysfunctional enzyme: build-up of its substrate and metabolites and a paucity of its product. OTC catalyzes the reaction between ornithine and carbamoyl phosphate to form citrulline and inorganic phosphate. The laboratory footprint of OTCD is low citrulline and elevated orotic acid due to funneling of excess carbamoyl phosphate into the pyrimidine synthesis pathway. Elevated uracil is also sensitive to OTCD and appears more useful than orotic acid in asymptomatic patients [23]. Orotic acid appears very sensitive to stress and is therefore a useful diagnostic test in the acute setting [24]. Another set of laboratory findings characteristic of the UCDs is respiratory alkalosis in the setting of HA [25]. Hyperventilation occurs in order to promote cerebral vasoconstriction and reduce cerebral edema likely secondary to the osmotic pressure exerted by the elevated glutamine levels. Additional diagnostic tests now less frequently used include measurement of orotodinuria after an allopurinol challenge [26] and measurement of urinary orotic acid after controlled protein loading [27].

### 4.5. Treatment

Acute decompensation should be treated immediately with hemodialysis or venovenous hemofiltration due to concern for cerebral edema. Medical treatment with intravenous “ammonia scavengers,” sodium benzoate, and sodium phenylacetate in 10% glucose solution is also warranted [28]. Benzoate conjugates with glycine to form hippuric acid, while phenylacetate binds glutamine. These nitrogen-containing compounds are both filtered and secreted in the kidney and allow for rapid excretion. Glucose supplementation is used to reduce the role of amino acid catabolism in cellular respiration. In our cases of protein/amino acid excess, this may not be as effective because there is no calorie deficit. Glucose metabolites still likely provide competitive inhibition slowing the rate of amino acid deamination. Supplemental L-arginine or citrulline is also given to improve downstream reaction kinetics and maximize urea production. Full treatment guidelines are discussed in the following review [28] and on the following link developed by the Urea Cycle Rare Disorders Consortium [29].

## 5. Conclusions

The differential for encephalopathy in an adult is broad and diagnosis is extremely difficult. In a patient with no previous medical history, a late-onset presentation of an inborn error of metabolism must be considered. The urea cycle disorders are prominent among this list and important to recognize due to the potentially fatal consequences of untreated HA. Recent increases in dietary protein intake have been increasingly identified as a trigger of HA in undiagnosed individuals with partial OTCD. Whether part of a low-carbohydrate high-protein diet, an effort to body build, or simply a gastronomic indulgence, increased protein consumption overwhelms partially functional enzymes and leads to elevated levels of ammonia and its metabolites. Thus, in patients with acute mental status changes, family history, dietary preferences, and recent diet changes may provide essential clues into the trigger of encephalopathy by suggesting for (or against) a defect in the urea cycle.

## Supplementary Materials

Supplementary Materials
*Supplementary Table 1*: supplemental case information.
