# Supplementary material for: Late Onset Ornithine Transcarbamylase Deficiency Triggered by an Acute Increase in Protein Intake: A Review of 10 Cases Reported in the Literature
Source: Case Rep Genet. 2020 Apr 25;2020:7024735. doi: 10.1155/2020/7024735 (PMC7197010; doi:10.1155/2020/7024735)
Supplement: Supplementary Materials — Supplementary Table 1: supplemental case information. [file 7024735.f1.pdf]

Supplemental Case Information

Case	Treatment	Labs (μM), Imaging, Sequencing	Outcome
1<br>Gaspari<br>2003	The patient was initially treated with 10 mg diazepam; the seizures stopped but the patient remained unresponsive. Based on history provided by family, a metabolic disorder was suspected and NH_3 level was measured. The patient was then given 8 mg/d lorazepam, lactulose, 10% glucose infusion, and discontinued protein intake. After 24 hours generalized seizures returned followed by coma (GCS 4) so patient transferred to ICU and started on mechanical ventilation. Day 5: given V-V hemodiafiltration (VVHDF), IV 10% Na benzoate and L-arginine-HCl with improvement. On day 7 regained consciousness and was discharged from ICU on day 10	NH_3 192, Repeat in ICU NH_3 576, post-VVHDF NH_3 27<br><br>Glutamine 1100, ornithine 18, arginine 22, citrulline 10<br><br>Urinary orotic acid 230 μmol/mmol Cr<br><br>EEG: slow waves, no epileptiform activity<br><br>CT: diffuse brain swelling with mild compression of lateral and 3rd ventricles<br><br>MRI: diffuse acute cortical swelling, most prominently insular and temporal regions	No neuro deficits at 1 year follow-up, patient refused intellectual testing
2<br>Panlaqui<br>2008	Given asterixis and elevated NH_3 the patient received continuous VVHDF, IV L-arginine HCl 10% 210 mg/kg/d, and protein free glucose polymer 15% (12 kcal/kg). An enteral protein restricted feed of 0.5 gm/kg was introduced in addition to glucose polymer.	(initial, day 3) NH_3 (390, 39), Glutamine (1300, 490), Citrulline (10, 4), Ornithine (26, 57), arginine (53, 180)<br><br>CT: normal<br><br>MRI: swelling with increased T2 signal in cortical gray matter, sparing perirolandic and occipital gyri<br><br>DNA: Arg40His (119G>A)	Generalized cognitive deficits at 6 months, expected to require support and rehab for foreseeable future

3<br>Ben-Ari<br>2010	The initial workup was negative and the patient became increasingly disoriented and progressed to seizures on day 4. At this point he was given valproate while EEG suggested a metabolic cause so NH_3 was measured. The patient deteriorated into coma and was transferred to ICU and mechanically ventilated. There patient received hemodialysis and was given L-arginine, Na phenylbutyrate, and IV 20% glucose/20% intralipid prep, while dietary protein eliminated was for 24 h. The NH_3 level normalized after two dialysis sessions and mental status recovered.	NH_3 386, glutamine 1381, citrulline 14, increased urinary orotic acid and uracil<br><br>Initial CT: normal<br><br>Initial MRI: normal<br><br>Initial EEG: normal<br><br>Day 4 EEG: generalized slowing with presence of triphasic waves and delta waves<br><br>Day 4 CT: cerebral edema<br><br>DNA: Ile159Met (477wobble>G)	Patient appeared active and healthy at follow-up a few months later
4<br>Thurlow<br>2010	Workup revealed elevated INR, HA, and respiratory alkalosis in the context of possible subarachnoid hemorrhage on CT. Consult physician advised team that HA was "red herring" so organic acid results were not processed urgently. The patient developed cerebral edema and seizures and became comatose with fixed, dilated pupils. Life support was withdrawn on day 4.	NH_3 348 pH 7.504<br><br>Urine orotic acid (223 µmol/mmol Cr), urine uracil (190 µmol/mmol Cr)<br><br>CT: possible subarachnoid bleed<br><br>Follow-up CT: no SAH visualized<br><br>DNA: Arg40Cys (118C>T)	Patient died on hospital day 4
5<br>ABC<br>2010	NA	NA	Patient died after "brief illness"
6<br>Telegraph<br>2010	The patient was admitted to ICU and mechanically ventilated. After 4 days in coma, an EEG and elevated NH_3 suggested metabolic etiology leading to appropriate labs and treatment for UCD diagnosis.	Elevated NH_3, elevated urinary orotic acid<br><br>EEG: "suggested ammonia poisoning"	Fully return to "normal brain function." Fully recovered
7<br>Choi<br>2012	HA was identified on preliminary labs. The patient was given lactulose enema but showed no clinical improvement and NH_3 continued to rise. Patient received acute HD during which he had generalized tonic-clonic seizure treated with IV lorazepam. He progressed to nonconvulsive status epilepticus treated with levetiracetam. NH_3 rose again post-HD leading to suspicion of UCD. Patient was given 3 g Arg and 3 g Na benzoate q4-6h with 10% dextrose and protein-free formula. He was dialyzed once more leading to NH_3 stabilization at <30. His mental status returned to normal after 5 days, and he was discharged home after 2 weeks	NH_3 143.8 Pre-HD 370 Post-HD 170, 228, 36, 107, <30<br><br>Ornithine 196, citrulline 3.<br><br>Elevated urinary orotic acid (603.5 mg/mg Cr) and mild uracil peak<br><br>CT: normal<br><br>MRI w/DWI: normal	Long-term function unclear

8<br>Rush<br>2014	HA was identified on admission labs, but was not treated and the patient developed cerebral edema and seizures. A metabolic specialist raised concern for UCD and IV Na phenylacetate/benzoate was started with normalization of NH_3. Confusion persisted for 1 week afterward.	NH_3 323, urinary orotic acid 3.6 mmol/mol Cr<br><br>DNA: Gly188Ala (563G>C)	Sustained moderate right-sided hearing loss, but otherwise made a full recovery and is in good health.
9<br>Alameri<br>2015	HA was identified on preliminary labs and patient was given lactulose without showing clinical improvement or fall in NH_3 level. On day 2 the patient was intubated, CT scan was repeated, and amino acid tests were ordered. The patient started on arginine, Na benzoate, and intermittent HD. The 3rd round of HD showed improved NH_3 levels but no change in consciousness. EEG showed generalized disturbance of cerebral activity.	NH_3 787, citrulline 7, urinary orotic acid 27.7 mmol/mol Cr<br><br>Initial CT: normal<br><br>Day 2 CT: diffuse edema<br><br>Initial MRI: normal<br><br>Initial EEG: mild diffuse slowing<br><br>Repeat EEG: severely attenuated, nonreactive<br><br>DNA: Arg40His (119G>A)	During recovery patient developed ventilator associated pneumonia, and severe C. difficile resulting in death
10<br>PerthNow<br>2017	A UCD was identified on hospital day 3.	NA	Patient pronounced brain-dead on hospital day 4

Normal values (expanded to include variation between labs): Ammonia (11-60 µM) Glutamine (337-700 µM), ornithine (20-125), arginine (54-130), citrulline (12-62), orotic acid (0-10), ornithine (19-81), urinary orotic acid (0-1.3 mmol/mol creatinine, <5µg/mg Cr) urinary uracil (<50 µmol/mmol Cr)
